# Association between the age-to-serum albumin ratio and all-cause mortality in patients with acute myocardial infarction: a retrospective cohort study

**DOI:** 10.3389/fcvm.2025.1667312

**Published:** 2025-11-25

**Authors:** Xue-Cheng Song, Yong Xia, Qiang Feng, Yong-Ming He

**Affiliations:** 1 Division of Cardiology, The First Affiliated Hospital of Soochow University, Suzhou, Jiangsu, China; 2 Division of Cardiology, The Third People's Hospital of Bengbu, Bengbu, Anhui, China; 3 Division of Cardiology, People's Hospital of Suzhou Taihu Lake National Tourism Resort, Suzhou, Jiangsu, China; 4 Division of Cardiology, Handan Central Hospital, Handan, Hebei, China

**Keywords:** age-to-serum albumin ratio, A2A index, all-cause mortality, acute myocardial infarction, risk stratification

## Abstract

**Background:**

Acute myocardial infarction (AMI) remains a predominant cause of cardiovascular death, necessitating accurate risk stratification. Existing risk scores like the ACEF (Age, Creatinine, Ejection Fraction) score and GRACE (Global Registry of Acute Coronary Events) score have limitations in complexity and subjectivity. This study aimed to investigate the novel age-to-serum albumin ratio (A2A Index) as a simple, objective predictive marker for all-cause mortality in AMI patients.

**Methods:**

The A2A Index was retrospectively calculated by dividing age by serum albumin in 1,007 consecutively enrolled AMI patients with 4-year median follow-up. The association between the A2A Index and all-cause mortality was assessed using Kaplan–Meier survival analysis, Cox regression analysis, and restricted cubic spline. The predictive performance of the A2A Index was compared with the ACEF and GRACE scores.

**Results:**

The A2A Index was capable of independently predicting all-cause mortality after multivariable adjustment [hazard ratio (HR) 4.98 per one-unit increase in A2A Index; 95% CI: 3.34–7.43; *P* < 0.001]. Restricted cubic splines illustrated a significant J-shaped dose-response relationship between the A2A Index and all-cause mortality risk (*P*-nonlinearity < 0.001). The A2A Index showed comparable discrimination to ACEF score [area under the curve (AUC): 0.83 vs. 0.83; *P* = 0.656] and superior to GRACE score (AUC: 0.83 vs. 0.80; *P* = 0.041), with a good calibration (*χ*^2^ = 9.08; *P* = 0.336). The optimal cutoff value for the A2A Index was 1.86, with a sensitivity of 79% and a specificity of 70%.

**Conclusion:**

The A2A Index is a simple and independent predictor of all-cause mortality in AMI patients, superior to GRACE score and comparable to ACEF score, with >1.86 indicating high mortality risk.

## 1 Introduction

Acute myocardial infarction (AMI) remains one of the leading causes of cardiovascular mortality worldwide (1, 2). Accurate and timely prognostic assessment is crucial for optimizing therapeutic decision-making and improving clinical outcomes.

Several risk stratification models, such as the GRACE (Global Registry of Acute Coronary Events), ACEF [Age, Creatinine, and Left Ventricular Ejection Fraction (LVEF)], and ACEF II scores, have been widely used and validated in clinical practice (3–7). However, these scoring systems may be constrained by underestimating risk, computational complexity, multi-parameter dependence, and subjective predictor interpretation (8–10). Although the ACEF score contains only three variables, its reliance on operator-dependent LVEF measurements remains problematic. Therefore, there is a need for an objective prognostic marker that is easily obtainable, requires few clinical parameters, and can be readily applied in routine clinical settings.

Serum albumin, accounting for approximately 50% of total plasma proteins (11), possesses anti-inflammatory, antioxidant, and antithrombotic properties (12–15). In recent years, it has been increasingly recognized as a protective factor in systemic inflammation (16, 17) and a powerful predictor of adverse cardiovascular outcomes (18, 19). A prospective cohort study (*n* = 4,947; median follow-up 4.42 years) revealed that lower serum albumin were associated with increased risks of hospitalization [hazard ratio (HR) = 1.58; 95% CI: 1.36–1.82; *P* < 0.001] and all-cause death (HR = 1.67; 95% CI: 1.24–2.24; *P* < 0.001) (20). Our research team first reported a dose–response relationship between low serum albumin levels and the risk of acute myocardial infarction in a Chinese Han population (HR = 1.79; 95% CI: 1.54–2.04) (21). Subsequent studies have further confirmed serum albumin as an independent prognostic biomarker for AMI, correlating with disease severity and adverse outcomes (22–25). Aging is associated with a progressive decline in albumin synthesis and increased inflammatory burden, which may synergistically worsen outcomes in ACS.

Based on this evidence, we proposed the age-to-serum albumin ratio (A2A Index) as a novel prognostic biomarker. The current study aimed to evaluate the association between the A2A Index and all-cause mortality in patients with acute myocardial infarction, which may serve as a practical adjunct in settings where GRACE score variables are unavailable or difficult to obtain rapidly.

## 2 Methods

### 2.1 Study participants

**Figure 1:**
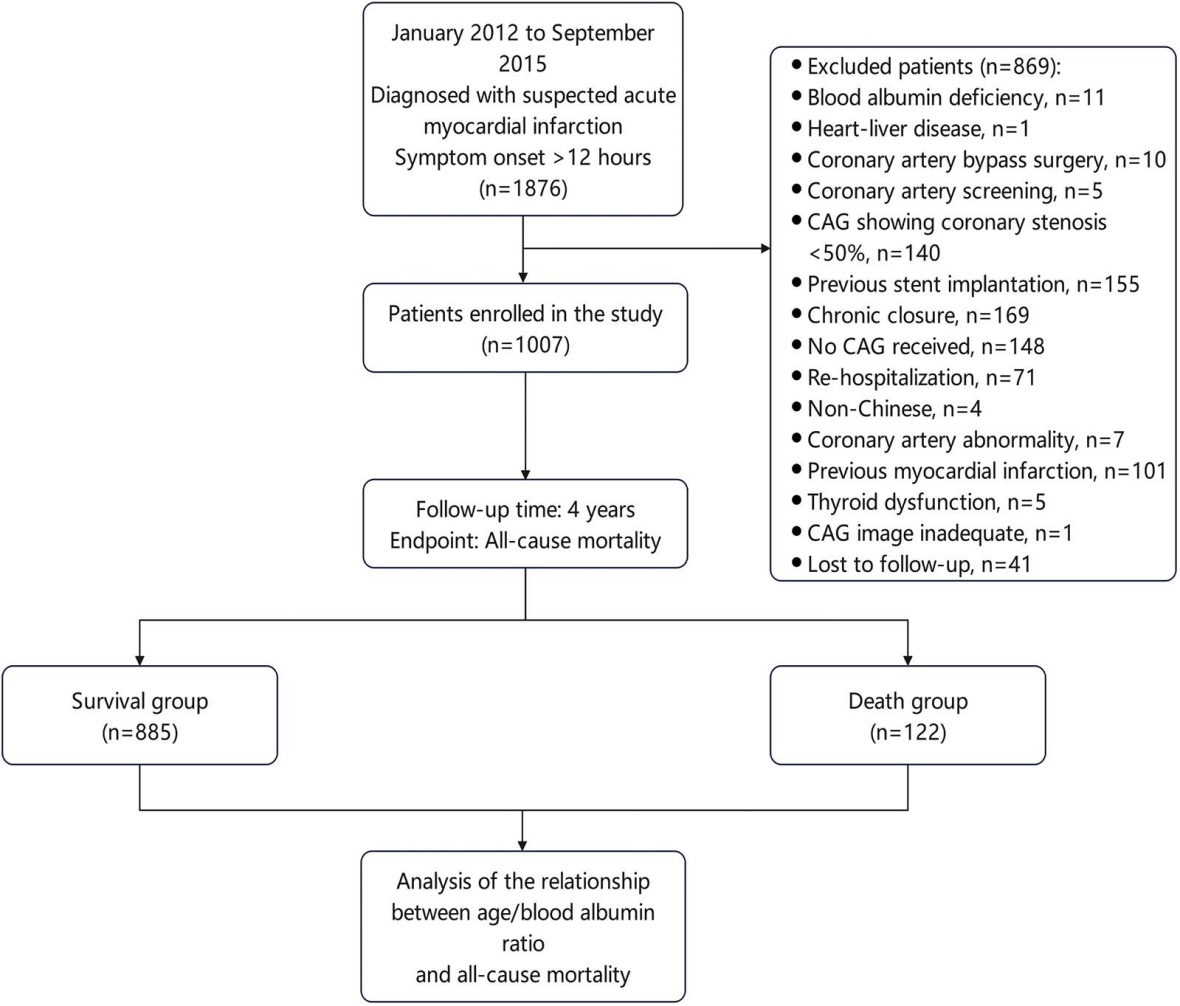
Flowchart for patient selection.

A total of 1,876 suspected AMI patients with high troponin I levels and chest pain lasting longer than 12 h, who were hospitalized to the First Affiliated Hospital of Soochow University's Department of Cardiology between January 2012 and September 2015 were retrospectively included in this study. 1,007 patients were finally included according to the inclusion criteria. They were divided into a death group (*n* = 122) and a survival group (*n* = 885) according to their survival status. Figure 1 displayed the inclusion and exclusion criteria (26).

### 2.2 Data collection

Clinical baseline data included: 1) Demographic characteristics: age, sex, and lifestyle; 2) Medical history: hypertension, diabetes mellitus, stroke, smoking, and alcohol consumption; 3) Height and weight; 4) ECG characteristics; 5) Coronary angiographic findings: single-vessel, double-vessel, or multi-vessel disease; 6) Whether PCI was performed; 7) Left ventricular ejection fraction; 8) Laboratory parameters: blood lipids, serum albumin, serum creatinine, blood glucose, and troponin I; 9) ACEF score and GRACE score.

Biochemical indicators were obtained from fasting blood samples collected on the second day after admission (usually after at least 8 h of fasting) and analyzed using a Siemens 2,400 fully automated biochemical analyzer (serum albumin was determined using the bromocresol green method). For each enrolled patient, risk scores were calculated: the GRACE score according to established methodology (27), and the ACEF score using the formula: age (years)/left ventricular ejection fraction (%)+creatinine score (1 if serum creatinine ≥2 mg/dL; 0 if <2 mg/dL) (28).

### 2.3 Definitions

All patients were followed up for 4 years after discharge, with the primary endpoint being all-cause mortality. All-cause mortality was defined according to the standardized Academic Research Consortium-2 (ARC-2) definition as death from any cause during the study period (29). Acute myocardial infarction (AMI) was defined according to the Third Universal Definition of Myocardial Infarction (2012) (30). The A2A Index (A2A Index) is defined as the age to serum albumin ratio, calculated as age (years)/serum albumin (g/L). We acknowledge that the GRACE score was primarily developed and validated for predicting in-hospital and 6-month post-discharge mortality. Nonetheless, our study aimed to evaluate the A2A index for long-term mortality (up to 4 years), a timeframe beyond the GRACE score's original design.

### 2.4 Candidate variables and risk models

Candidate variables identified by univariate analysis (*P* < 0.05) were incorporated into multivariate Cox proportional hazards models. The variance inflation factors (VIFs) of all included variables were below 5, suggesting no significant multicollinearity.

Three risk models were defined: Model 1: univariate analysis; Model 2: adjusted for sex, body mass index (BMI), smoking status, and alcohol consumption; Model 3: adjusted for sex, BMI, smoking, alcohol consumption, triglycerides, troponin I, serum creatinine, left ventricular ejection fraction (LVEF), hypertension, diabetes mellitus, prior stroke, number of diseased coronary vessels, and percutaneous coronary intervention (PCI).

### 2.5 Statistical analysis

Statistical analysis was performed using Stata/MP 17.0 (Stata Corp, Texas, USA). Missing values were handled using multiple imputation (chained equations, 25 iterations). Continuous variables were expressed as mean ± standard deviation or median (interquartile range, IQR), while categorical variables were expressed as frequencies and percentages. Comparisons were performed using the Kruskal–Wallis rank test or Pearson's chi-square test, as appropriate. Kaplan–Meier curves were used to compare survival differences between different A2A Index groups. Candidate variables identified by univariate analysis (*P-*value <0.05) were incorporated into multivariate Cox proportional hazards model to assess the independent predictive value of the A2A Index for all-cause mortality. Restricted cubic spline was used to analyze non-linear relationships between the A2A Index and all-cause mortality. Subgroup analysis and interaction testing evaluated the consistency of the relationship between the A2A Index and all-cause mortality across different population characteristics. Sensitivity analysis assessed the robustness of results by excluding specific samples. Receiver operating characteristic (ROC) curves were used to evaluate the discriminative ability of the A2A Index compared to ACEF score and GRACE score, while the Hosmer–Lemeshow test assessed the calibration. We have also performed time-dependent ROC analysis and Landmark analysis. Comparative analysis of different ROC curves using DeLong test. All tests were two-sided, a *P-*value <0.05 was considered statistically significant.

## 3 Results

### 3.1 Baseline characteristics

Baseline clinical characteristics were presented in Table 1. Compared with the survival group, patients in the death group were older (median age 75 vs. 65 years), had higher serum creatinine levels and A2A Index values, and exhibited a higher prevalence of hypertension, diabetes mellitus, or prior stroke (all *P* < 0.05). In contrast, serum albumin levels and left ventricular ejection fraction (LVEF) were significantly lower in the death group (*P* < 0.05). Unexpectedly, the proportions of both smoking and drinking in the death group were low. The A2A Index was left-skewed in distribution with a smaller proportion exhibiting extremely high values associated with poor prognosis.

**Table 1:** Clinical baseline data.

Factors	Missing *n* (%)	Survival group	Death group	*P*
		*N* = 885	*N* = 122	
Age (years)		65.00 (56.00–72.00)	75.00 (68.00–80.00)	<0.001
Sex *n* (%)				<0.001
Female		169 (19.10)	42 (34.43)	
Male		716 (80.90)	80 (65.57)	
Height (cm)	19 (1.89)	167.00 (160.00–170.00)	162.00 (156.00–168.00)	<0.001
Weight (kg)	132 (13.11)	66.00 (60.00–75.00)	64.00 (54.00–70.00)	<0.001
BMI (kg/m^2^)		24.09 (21.88–26.30)	23.14 (21.63–24.80)	0.026
STEMI *n* (%)		438 (49.49)	61 (50.00)	0.920
Hypertension *n* (%)		568 (64.18)	99 (81.15)	<0.001
Type 2 diabetes *n* (%)		189 (21.36)	46 (37.70)	<0.001
Stroke *n* (%)		46 (5.20)	13 (10.66)	0.016
Smoking *n* (%)				<0.001
Never		311 (35.14)	64 (52.46)	
Past		84 (9.49)	16 (13.11)	
Current		490 (55.37)	42 (34.43)	
Alcohol intake *n* (%)				<0.001
Never		636 (71.86)	106 (86.89)	
Past		29 (3.28)	6 (4.92)	
Current		220 (24.86)	10 (8.20)	
PCI treatment *n* (%)		833 (94.12)	97 (79.51)	<0.001
Diseased vessels *n* (%)				<0.001
No		7 (0.79)	1 (0.82)	
One vessel		532 (60.11)	49 (40.16)	
Two vessels		256 (28.93)	47 (38.52)	
Three vessels		90 (10.17)	25 (20.49)	
LDL-c, mmol/L	4 (0.40)	2.51 (2.10–3.13)	2.24 (1.68–3.07)	0.014
HDL-c, mmol/L	4 (0.40)	0.95 (0.90–1.11)	0.93 (0.88–1.14)	0.420
Triglyceride, mmol/L	2 (0.20)	1.26 (0.91–1.80)	0.93 (0.73–1.34)	<0.001
TC, mmol/L		4.02 (3.39–4.77)	3.77 (3.04–4.60)	0.017
Serum albumin, g/L		39.00 (36.10–41.40)	35.10 (32.00–37.70)	<0.001
LP(a), mg/dL	61 (6.06)	110.00 (49.50–251.00)	108.00 (57.00–273.00)	0.24
Troponin I, ng/mL	4 (0.40)	6.53 (1.69–22.60)	7.16 (1.24–50.60)	0.23
Creatinine, μmol/L		70.80 (61.00–82.60)	81.85 (65.00–105.70)	<0.001
Glucose, mmol/L		5.38 (4.83–6.30)	5.59 (4.78–6.91)	0.29
Ejection fraction, %	53 (5.26)	55.00 (45.00–63.00)	43.00 (36.00–53.00)	<0.001
ACEF score		1.20 (0.95–1.48)	1.77 (1.42–2.09)	<0.001
GRACE score		140.00 (117.00–162.00)	166.00 (151.00–198.00)	<0.001
A2A Index		1.65(1.38–1.93)	2.17(1.87–2.41)	<0.001

BMI, body mass index; STEMI, ST-Segment Elevation Myocardial Infarction; PCI, Percutaneous Coronary Intervention; LDL-c, low density lipoprotein cholesterol; HDL-c, high density lipoprotein cholesterol; TC, total cholesterol; LP(a), lipoprotein(a); ACEF, Age, Creatinine, Ejection Fraction; GRACE, Global Registry of Acute Coronary Events; A2A Index, age-to-serum albumin ratio. Continuous variables are presented as median (interquartile range) due to non-normal distribution; categorical variables are expressed as counts (percentages).

### 3.2 Univariate-and multivariable-adjusted associations between the A2A index and all-cause mortality (categorical scale)

**Figure 2:**
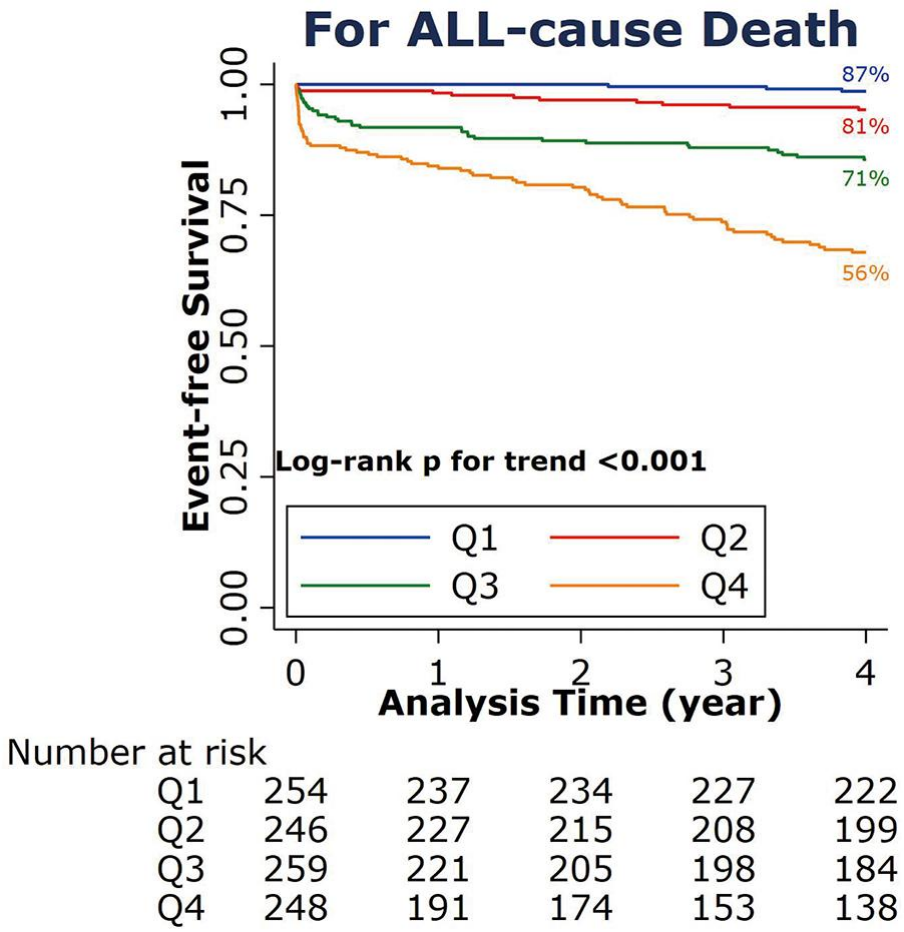
Kaplan–Meier survival curve for all-cause death at 4 years according to the A2A Index quartiles. A2A Index: age-to-serum albumin ratio.

The A2A Index was stratified into four quartiles: Q1 (<1.43), Q2 (1.43–1.69), Q3 (1.70–2.02), and Q4 (≥2.02). Correlation analysis indicated a moderate positive association between the A2A Index and the risk of all-cause mortality (Somers' D = 0.55; 95% CI: 0.475–0.622; *P* < 0.001). As shown in (Figure 2), Kaplan–Meier survival declined with increasing A2A quartiles. Higher A2A quartiles were associated with progressively lower survival probabilities (log-rank *P* < 0.001), with the Q4 group exhibiting the lowest 4-year survival rate at 56%.

The results of the univariate analysis and multivariate Cox proportional hazards regression were presented in Table 2. Using the lowest A2A quartile (Q1) as the reference, the adjusted hazard ratios (HRs) for all-cause mortality were 5.71 (95% CI: 1.40–23.36) in Q2, 18.59 (95% CI: 4.59–73.39) in Q3, and 27.64 (95% CI: 6.99–109.18) in Q4, demonstrating a significant dose–response effect (*P* for trend <0.001). we have performed a landmark analysis excluding 6 events occurring within the first 48 h. The results consistently demonstrate that higher A2A quartiles were associated with progressively lower survival probabilities (log-rank *P* < 0.001) (Supplementary Figure 3A).

**Table 2:** Association between A2A Index quartile groups and all-cause mortality.

A2A index quartiles	Q1 (reference)	Q2	Q3	Q4	*P* for trend
		HR (95% CI)	HR (95% CI)	HR (95% CI)	
Model 1	1	3.91 (1.09–14.00)	12.62 (3.88–41.02)	30.70 (9.67–97.42)	<0.001
Model 2	1	3.80 (1.06–13.65)	12.14 (3.69–39.87)	28.06 (8.71–90.44)	<0.001
Model 3	1	5.71 (1.40–23.36)	18.59 (4.59–73.39)	27.64 (6.99–109.18)	<0.001

A2A = age-to-serum albumin ratio. Model 1: univariate analysis. Model 2: adjusted for sex, body mass index (BMI), smoking status, and alcohol consumption. Model 3: adjusted for sex, BMI, smoking, alcohol consumption, triglycerides, troponin I, serum creatinine, left ventricular ejection fraction (LVEF), hypertension, diabetes mellitus, prior stroke, number of diseased coronary vessels, and percutaneous coronary intervention (PCI). HR, hazard ratio; CI, confidence interval.

### 3.3 Univariate-and multivariable-adjusted associations between the A2A index and all-cause mortality (continuous scale)

The A2A Index was analyzed as a continuous variable in univariate analysis (Table 3), each one-unit increase in the A2A Index was associated with a 7.62-fold higher risk of all-cause mortality (HR = 7.62; 95% CI: 5.43–10.68; *P* < 0.001). In sex-stratified univariate analyses, the association was significant in both males (HR = 6.40; 95% CI: 4.34–10.16) and females (HR = 8.96; 95% CI: 4.90–16.36), with all *P*-values < 0.001. Multivariate Cox proportional hazards regression analysis showed that each one-unit increase of A2A Index was associated with a 4.98-fold higher risk of all-cause mortality (HR = 4.98; 95% CI: 3.34–7.43; *P* < 0.001) (Table 3).

**Table 3:** Association between A2A Index and all-cause mortality in different models.

Groups	Total		Male		Female	
	HR (95% CI)	*P* for trend	HR (95% CI)	*P* for trend	HR (95% CI)	*P* for trend
Model 1	7.62 (5.433–10.677)	<0.001	6.40 (4.342–10.157)	<0.001	8.96 (4.904–16.364)	<0.001
Model 2	7.30 (5.123–10.405)	<0.001	6.34 (4.230–10.417)	<0.001	9.24 (4.936–17.293)	<0.001
Model 3	4.98 (3.342–7.432)	<0.001	4.28 (2.620–6.980)	<0.001	6.58 (2.802–15.429)	<0.001

Model 1: univariate analysis. Model 2: adjusted for sex, body mass index (BMI), smoking status, and alcohol consumption. Model 3: adjusted for sex, BMI, smoking, alcohol consumption, triglycerides, troponin I, serum creatinine, left ventricular ejection fraction (LVEF), hypertension, diabetes mellitus, prior stroke, number of diseased coronary vessels, and receipt of percutaneous coronary intervention (PCI). HR, hazard ratio; CI, confidence interval.

**Figure 3:**
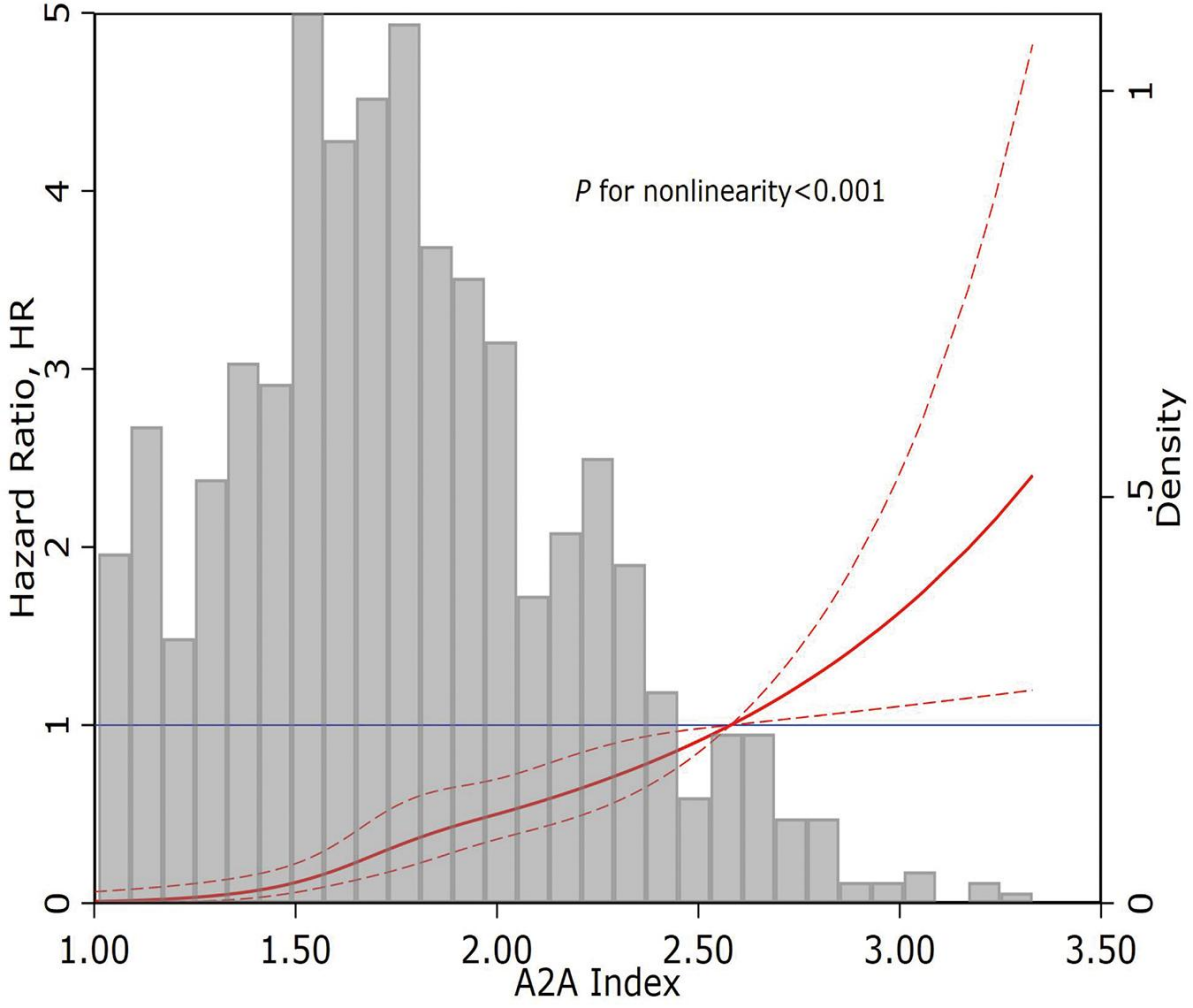
Restricted cubic spline curve of the A2A Index and all-cause mortality. A2A Index: age-to-serum albumin ratio.

Restricted cubic spline regression illustrated a significant J-shaped dose-response relationship between the A2A Index and all-cause mortality risk (*P*-nonlinearity < 0.001) (Figure 3).

### 3.4 Prediction performance of the A2A index for all-cause mortality: discrimination and calibration

**Figure 4:**
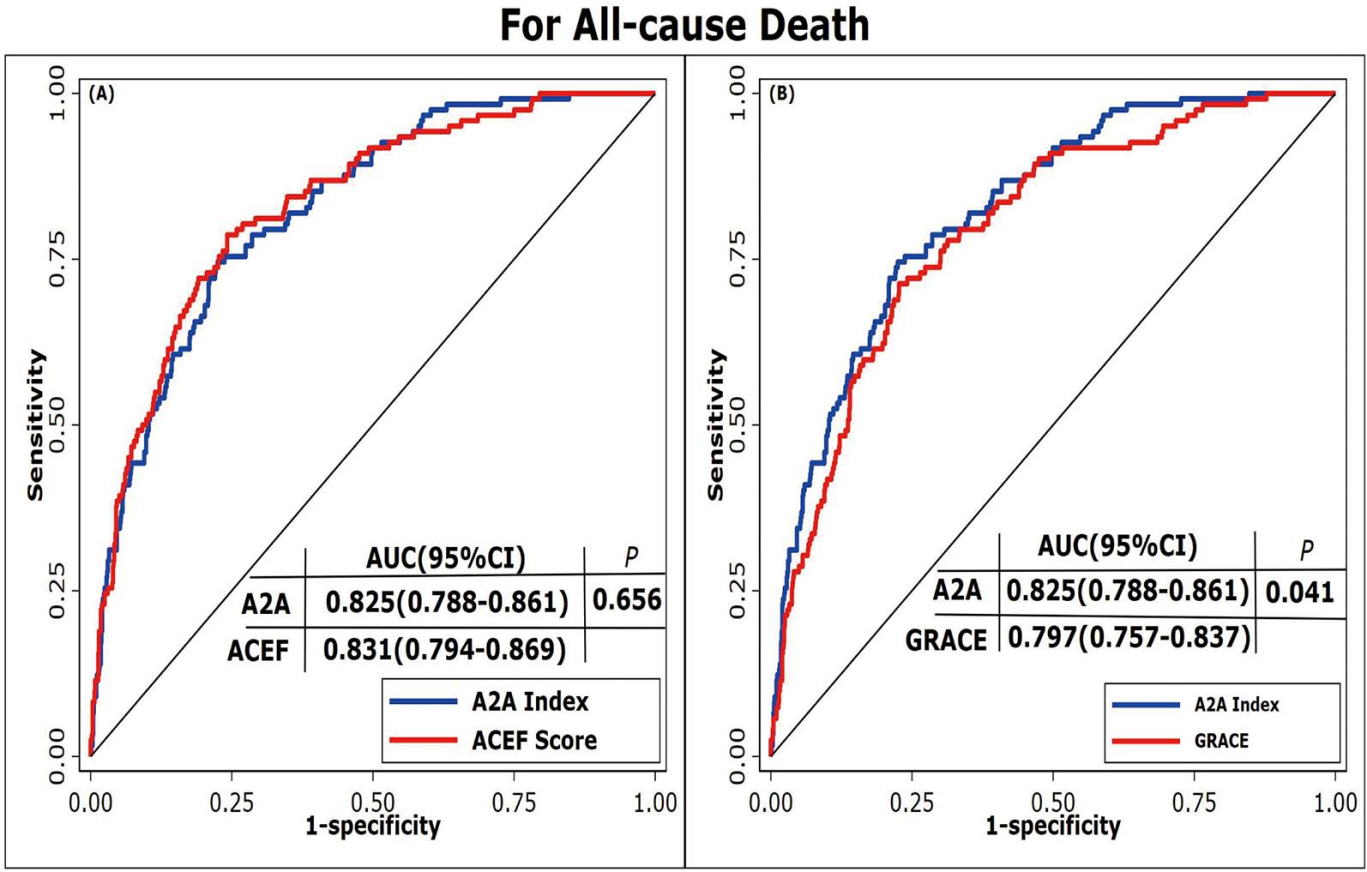
Receiver operating characteristic curves of different prediction models. **(A)** ROC curves comparing the A2A Index and ACEF score. **(B)** ROC curves comparing the A2A Index and GRACE score. A2A Index: age-to-serum albumin ratio; AUC: area under the curve; ACEF: Age, Creatinine, Ejection Fraction; GRACE: Global Registry of Acute Coronary Events.

**Figure 5:**
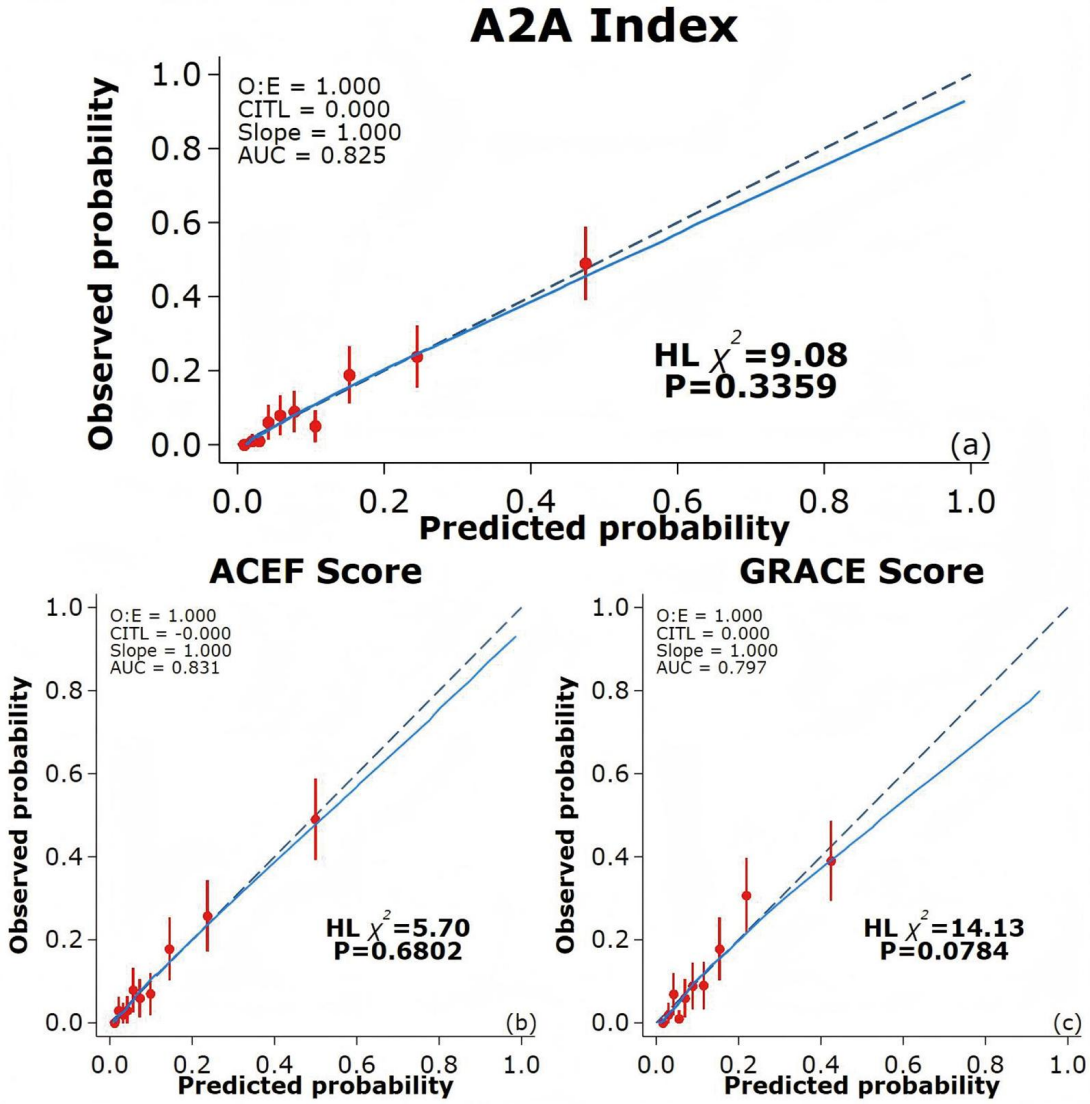
Calibration curves of different prediction models. **(a)** Calibration curve for the A2A Index; **(b)** Calibration curve for the ACEF score; **(c)** Calibration curve for the GRACE score. The blue solid line indicates the fitted curve, while the dashed diagonal line represents perfect calibration (slope = 1). CITL: calibration in the large; AUC: area under the curve; ACEF: Age, Creatinine, Ejection Fraction; GRACE: Global Registry of Acute Coronary Events.

ROC curves were used to evaluate the prediction performance of the A2A Index. The area under the curve (AUC) for the A2A Index was 0.83(95% CI: 0.79–0.86), which was significantly greater than that of the GRACE score [AUC = 0.80(95% CI: 0.76–0.84); *P* = 0.041] and comparable to the ACEF score [AUC = 0.83(95% CI: 0.79–0.87); *P* = 0.656] (Figure 4). The optimal cutoff value for the A2A Index was 1.86, with a sensitivity of 79% and a specificity of 70%. The A2A Index (*χ*^2^ = 9.08, *P* = 0.336) demonstrated a better calibration than the GRACE score (*χ*^2^ = 14.13, *P* = 0.078) and a comparable one to the ACEF score (*χ*^2^ = 5.70, *P* = 0.680) (Figure 5). we also performed time-dependent ROC analysis at 1, 2, 3and 4 years post-discharge. The results consistently demonstrate that the A2A index maintains discriminative ability over time, with AUC values of 0.821, 0.818, 0.831and 0.836 respectively. Within the initial two years, A2A index and GRACE score show equivalent risk; thereafter, A2A index is associated with a higher all-cause mortality risk than GRACE score (Supplementary Figures 1a–d and 2a).

### 3.5 Subgroup and sensitivity analyses

**Figure 6:**
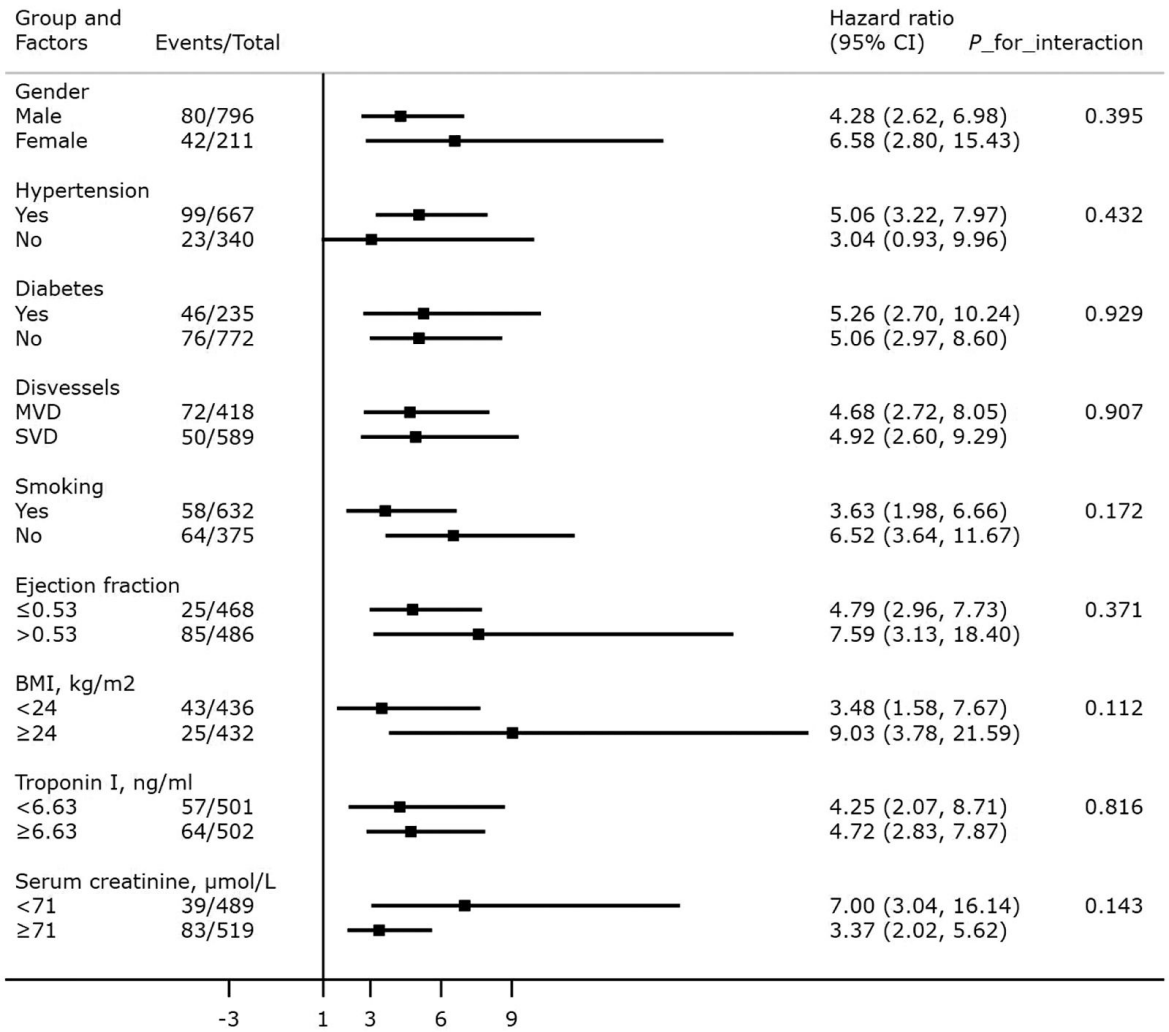
Association between the A2A Index and all-cause mortality: subgroup and interaction analyses. BMI: body mass index; SVD: single-vessel disease; MVD: multivessel disease.

After adjusting for confounding factors, an elevated A2A Index was significantly associated with increased mortality across all subgroups (*P* < 0.001), except among patients without hypertension (*P* > 0.05). No significant interactions were observed between the A2A Index and any subgroup variables (all *P* for interaction > 0.05). Detailed results of the subgroup analyses and interaction terms are shown in Figure 6.

Sensitivity analyses confirmed the robustness of the findings. The association between the A2A Index and all-cause mortality remained statistically significant after excluding patients who died within the first year of follow-up, those with missing BMI data, and those meeting both conditions (all *P* < 0.001). Data were shown in Supplementary Materials.

## 4 Discussion

This study yielded several key findings: (1) the age-to-serum albumin ratio (A2A Index) was first proposed as a strong and independent predictor of all-cause mortality in patients with acute myocardial infarction (AMI); (2) the A2A Index demonstrated a significant non-linear, J-shaped association with mortality risk, with a critical threshold at 1.86, beyond which high risk of mortality in patients with AMI; (3) the A2A Index outperformed the GRACE score and was comparable to the ACEF score, demonstrating excellent discrimination and calibration.

We acknowledge that the GRACE score remains the guideline-endorsed standard for risk stratification in ACS. We carefully avoid claims of outright superiority for the A2A index. Instead, we position it as a potential complementary tool that may add value in specific scenarios, such as in resource-limited settings where rapid, initial triage is needed or when certain GRACE variables are temporarily unavailable. The observed “smoking and alcohol paradox” in our study—where the survival group exhibited higher proportions of smokers (55.37% vs. 34.43%) and alcohol consumers (24.86% vs. 8.20%) compared to the mortality group—likely reflects a “healthy smoker/drinker bias.” This phenomenon suggests that high-risk behaviors exert an earlier selective pressure on individuals with weaker constitutions, resulting in long-term smokers/drinkers in the survival group possessing greater intrinsic compensatory capacity and tolerance. Indeed, the survival of these patients should not be attributed to their smoking or drinking behaviors; rather, it inversely indicates that they may possess certain protective factors or superior baseline health status. This manifests statistically as an association contrary to expected outcomes, underscoring the importance of considering selection bias when interpreting lifestyle factors in cardiovascular mortality studies.

The A2A Index combines two readily obtainable and objective variables with minimal subjective bias. Age serves as a fundamental predictor in cardiovascular mortality, with virtually all prognostic models (ACEF, GRACE, SYNTAXII) incorporating it as a key component. As the critical indicator of physiological reserve and cardiovascular deterioration, age was confirmed to be an independent predictor of mortality in GISSI-2 trial, with an odds ratio (OR) of 2.2 (95% CI: 1.60–2.90) for patients 61–70 years old and 3.9 (95% CI: 2.90–5.30) for patients >70 years old (31). Mehta et al. reported a significant age-dependent increase in 30-day mortality in a cohort study of 163,140 patients (65–69 years old: 10.9%, 70–74: 14.1%, 75–79: 18.5%, 80–84: 23.2%, >85: 31.2%, *P* = 0.001) (32). Serum albumin provides vascular protection through anti-inflammatory, antioxidant, and antithrombotic mechanisms, while its deficiency induces endothelial dysfunction and prothrombotic states (33, 34). Our previous research demonstrated a dose-response relationship between low albumin and cardiovascular mortality (21), with recent reviews confirming albumin as an independent predictor of both short and long-term mortality in AMI patients (35). Despite its established value, only the BACEF score systematically incorporates albumin among contemporary acute coronary syndrome (ACS) risk stratification tools (36). By integrating these parameters as a new biomarker, our study not only elucidates their dynamic interaction but also maintains predictive accuracy and clinical applicability, particularly valuable for emergency and resource-limited clinical settings.

In our cohort of 1,007 hospitalized AMI patients with 4-year follow-up, each 1-unit increase in the A2A Index was independently associated with higher all-cause mortality risk (adjusted HR = 4.92; 95% CI: 3.15–7.68; *P* < 0.001), after adjustment for 13 confounding variables including sex, BMI, lipid profile, renal function, and LVEF. Sensitivity analyses confirmed the robustness of this association, and subgroup analyses showed consistent effects across strata, with no significant interactions. Restricted cubic spline and Kaplan–Meier analyses revealed a J-shaped non-linear association between the A2A Index and mortality risk. Below 1.86, the risk remained stable but increased steeply beyond this threshold, indicating a pathophysiological decompensation threshold at which the combined impact of advanced age and hypoalbuminemia sharply elevates mortality risk. This non-linear behavior may better reflect the true biological trajectory than the linear age/LVEF ratio used in the ACEF score, enabling more accurate identification of high-risk patients and optimal timing for intervention. Despite comprising only two variables, the A2A Index demonstrated superior predictive performance to the GRACE score and was statistically equivalent to the ACEF score. ROC analysis showed that the AUC of A2A (0.825) was significantly greater than that of GRACE score (0.797, *P* = 0.041) and comparable to ACEF score (0.831, *P* = 0.656). The Hosmer–Lemeshow goodness-of-fit test also demonstrated better calibration for A2A (*χ*^2^ = 9.08; *P* = 0.336) than for GRACE score (*χ*^2^ = 14.13; *P* = 0.078). Unlike GRACE score, which incorporates potentially subjective parameters (e.g., Killip class, blood pressure, and heart rate), the A2A Index is derived solely from objective variables (age and serum albumin), effectively eliminating operator-related bias.

The role of albumin not merely as a nutritional marker but as a multifunctional protein with antioxidant, anti-inflammatory, and endothelial-stabilizing properties. The hypoalbuminemia in ACS reflects a confluence of systemic inflammation, oxidative stress, and endothelial dysfunction. Age serves as a key proxy for several high-risk physiological states in ACS. It reflects diminished physiological reserve, immunosenescence, and the cumulative burden of comorbidities, which collectively heighten vulnerability to the pathophysiological insults of acute coronary syndrome. The A2A index will combine and amplify the effects of the albumin and age. we explain that the A2A index is not intended to replace GRACE but could alter clinical practice by:(1) Simplifying Initial Triage: Serving as a rapid “risk flag” at admission or within the first 48 h, potentially identifying high-risk patients earlier in the care pathway.(2) Informing Monitoring Intensity: Its dynamic nature (e.g., Day-2 value) could help in serial risk assessment, guiding decisions on the intensity of monitoring or the urgency of further interventions.(3) Resource-Limited Application: Highlighting its utility in environments where calculating the full GRACE score is impractical, thus making risk stratification more accessible.

### 4.2 Limitations

This study has several limitations. First, it was a retrospective analysis conducted at a single center, and external validation using multicenter prospective cohorts is needed. Moreover, as the study population included only AMI patients who presented more than 12 h after symptom onset, the findings may not be generalizable to those receiving early interventional therapy. Second, only all-cause mortality was assessed as the clinical endpoint. Future research should incorporate specific cardiovascular outcomes to better evaluate the prognostic value of the A2A Index. Third, the A2A Index was calculated based solely on baseline measurements at admission, without accounting for dynamic changes in serum albumin levels during hospitalization or follow-up. Fourth, bromocresol Green Method (BCG) may overestimate certain parameters compared to direct biochemical assays. We will conduct further studies to externally validate our findings using alternative measurement techniques and diverse populations. Fifth, we must consider potential biases, such as those arising from measurement bias, unmeasured confounders, and sample selection bias.

## 5 Conclusions

The A2A Index is a simple, independent, and non-linear predictor of all-cause mortality in AMI patients, demonstrating superior performance to GRACE score and comparable utility to ACEF score. The A2A Index has a critical threshold at 1.86, beyond which the mortality risk increases sharply.

## Data Availability

The original contributions presented in the study are included in the article/Supplementary Material, further inquiries can be directed to the corresponding author.
