# Supplementary material for: Association between the age-to-serum albumin ratio and all-cause mortality in patients with acute myocardial infarction: a retrospective cohort study
Source: Front Cardiovasc Med. 2025 Nov 25;12:1667312. doi: 10.3389/fcvm.2025.1667312 (PMC12685903; doi:10.3389/fcvm.2025.1667312)
Supplement: Supplementary file 1 [file Datasheet1.pdf]

Supplementary file

Association Between the Age-to-Serum Albumin Ratio and All-Cause Mortality in Patients with Acute Myocardial Infarction: A Retrospective Cohort Study

Xue-Cheng Song†, Yong Xia†, Qiang Feng and Yong-Ming He*

Division of Cardiology, The First Affiliated Hospital of Soochow University, Suzhou, Jiangsu, 215006, China.

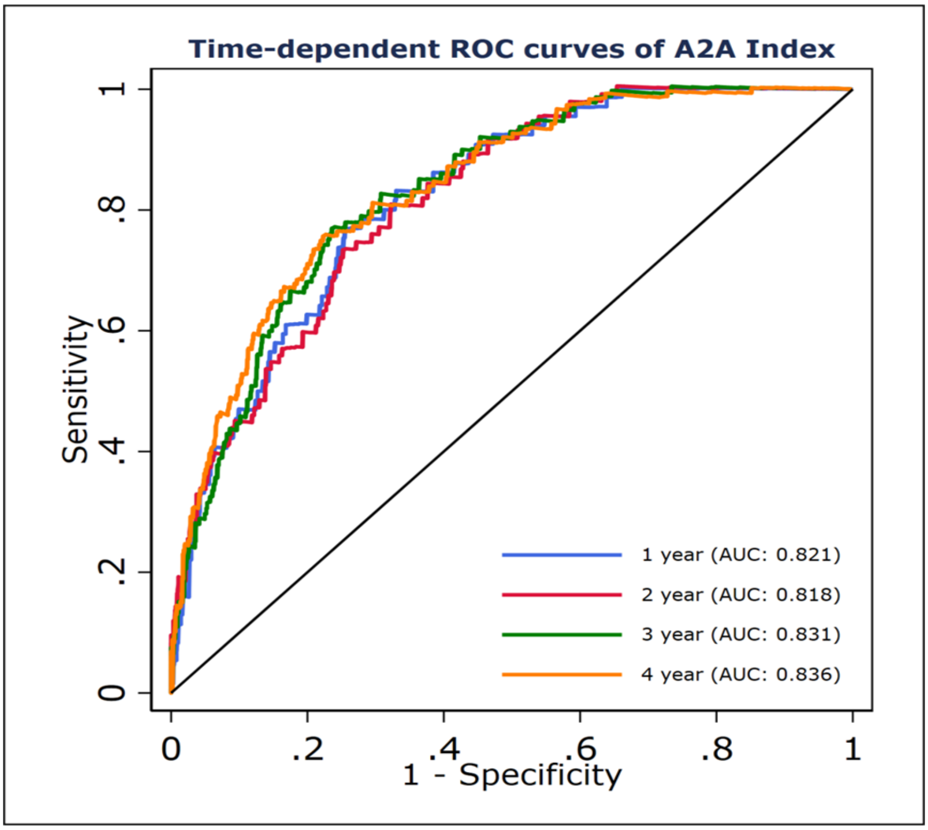

Supplementary Figure 1a. Time-dependent ROC curves of A2A Index.

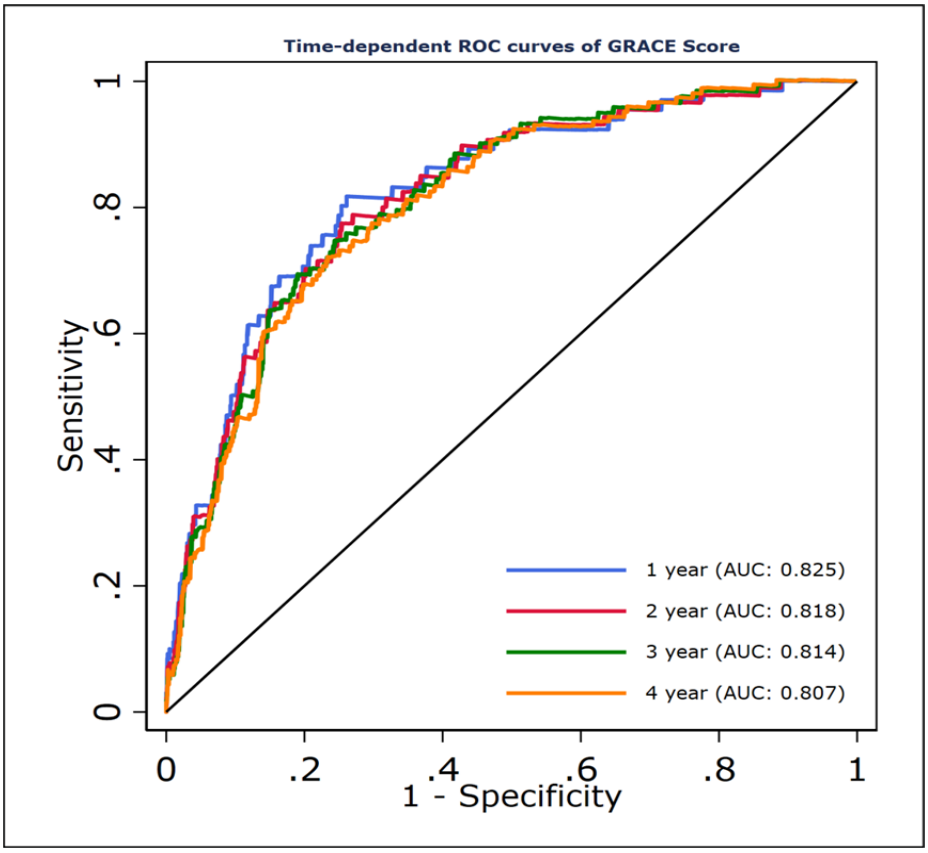

Supplementary Figure 1b. Time-dependent ROC curves of GRACE Score.

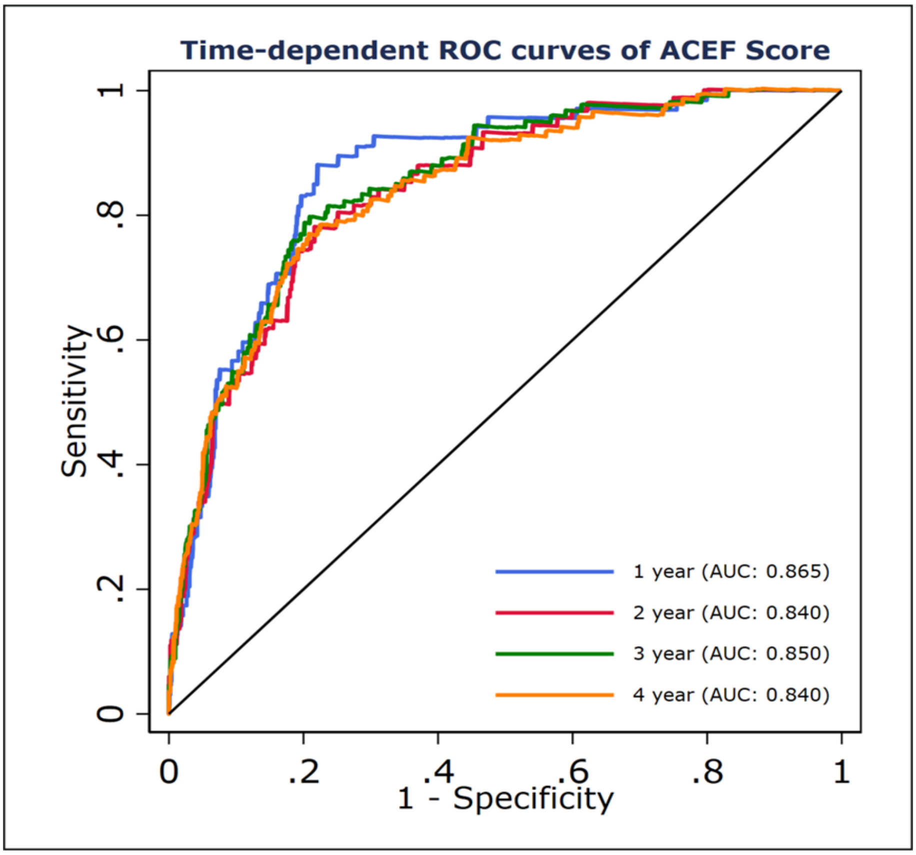

Supplementary Figure 1c. Time-dependent ROC curves of ACEF Score.

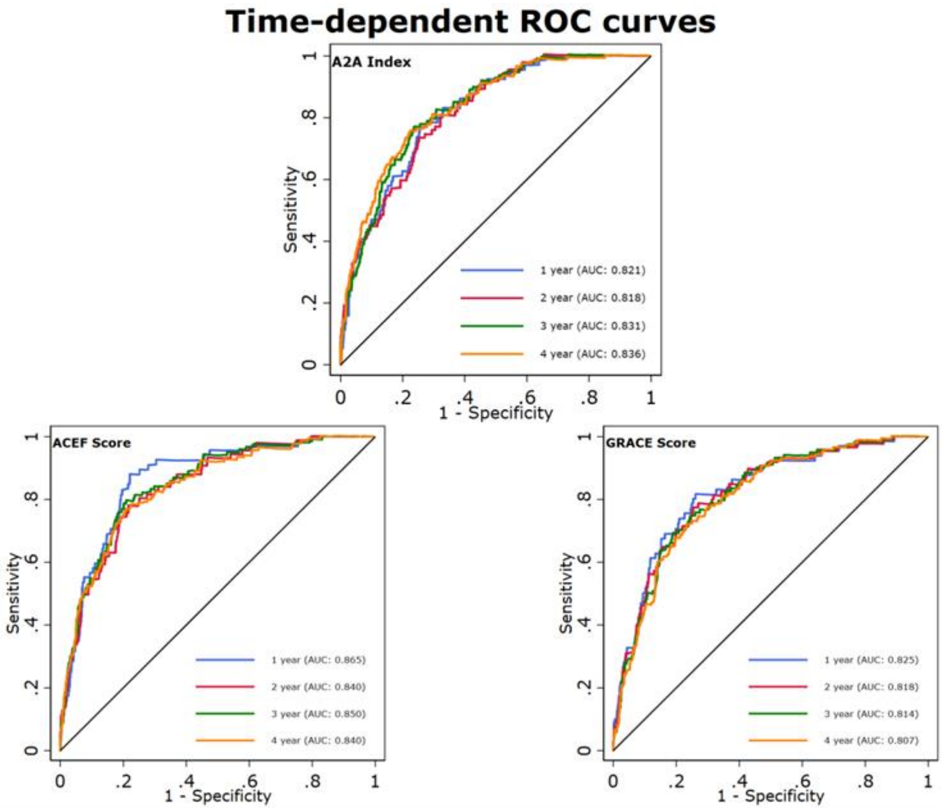

Supplementary Figure 1d. Time-dependent ROC curves of A2A Index comparing with GRACE Score

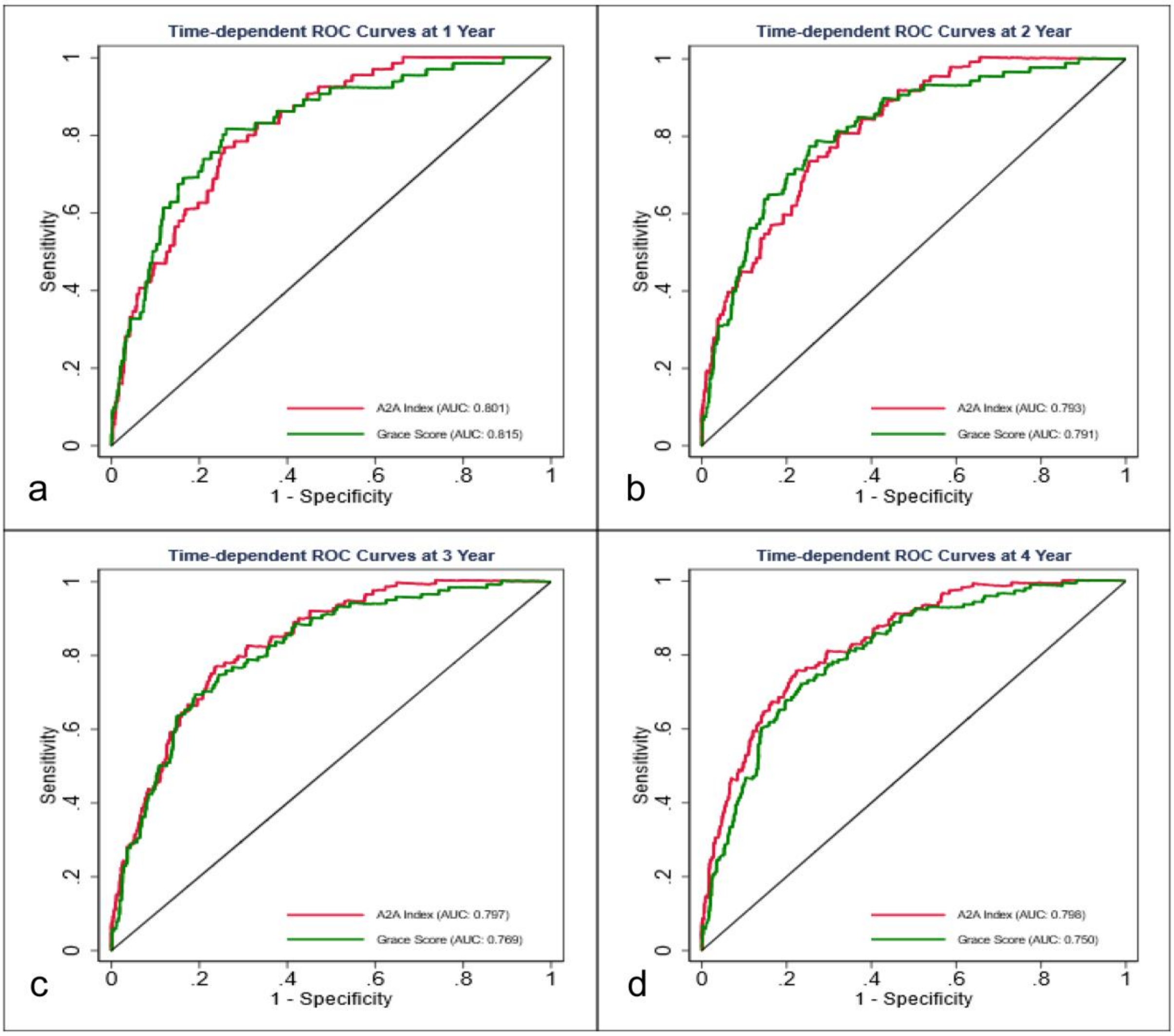

Supplementary Figure 2. Comparative analysis of ROC curves for A2A versus grace scores at 1- to 4-year follow-up using DeLong test.

Supplementary Table 2. The results of DeLong test

Year	AUC		DeLong Test
	A2A Index	Grace Score	
1	0.801	0.815	P=0.588
2	0.793	0.791	P=0.952
3	0.797	0.769	P=0.200

4	0.798	0.750	P=0.013

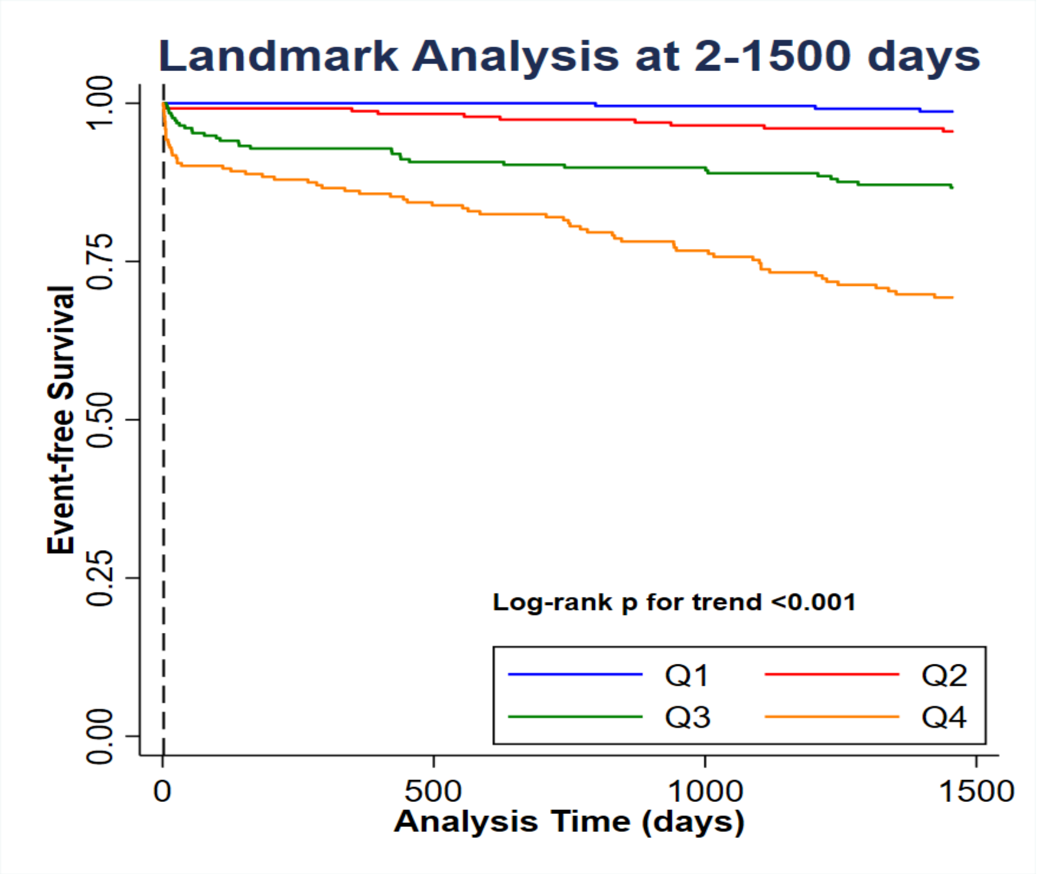

Supplementary Figure 3. landmark analysis excluding events occurring within the 2 days
