# Supplementary material for: Association between the age-to-serum albumin ratio and all-cause mortality in patients with acute myocardial infarction: a retrospective cohort study
Source: Front Cardiovasc Med. 2025 Nov 25;12:1667312. doi: 10.3389/fcvm.2025.1667312 (PMC12685903; doi:10.3389/fcvm.2025.1667312)
Supplement: Supplementary file 2 [file Table1.pdf]

# Supplementary file

**Association Between the Age-to-Serum Albumin Ratio and All-Cause Mortality in Patients with Acute Myocardial Infarction: A Retrospective Cohort Study**

Xue-Cheng Song†, Yong Xia†, Qiang Feng and Yong-Ming He*

Division of Cardiology, The First Affiliated Hospital of Soochow University, Suzhou, Jiangsu, 215006, China.

## Sensitivity analysis

**Supplementary Table 1. Association between A2A Index and all-cause mortality in populations with different characteristics**

| Groups | HR (95%CI) | *P* value |
|---|---|---|
| A (n=875) | 6.06 (3.07-11.97) | < 0.001 |
| B (n=868) | 4.85 (2.77-8.51) | < 0.001 |
| C (n=769) | 5.84(2.44-13.99) | < 0.001 |

Note: **(A):** Exclusion: death within the first year; **(B):** Exclusion: missing BMI; **(C):** Exclusion: death within the first year and missing BMI. Adjustment factors: sex, BMI, smoking, alcohol consumption, triglycerides, troponin I, serum creatinine, left ventricular ejection fraction (LVEF), hypertension, diabetes mellitus, prior stroke, number of diseased coronary vessels, and receipt of percutaneous coronary intervention (PCI). HR = hazard ratio; CI = confidence interval; BMI: body mass index; A2A Index: age-to-serum albumin ratio.
